# A Low-Carbohydrate Diet Realizes Medication Withdrawal: A Possible Opportunity for Effective Glycemic Control

**DOI:** 10.3389/fendo.2021.779636

**Published:** 2021-12-14

**Authors:** Yuxin Han, Bingfei Cheng, Yanjun Guo, Qing Wang, Nailong Yang, Peng Lin

**Affiliations:** ^1^ Department of Endocrinology and Metabolism, Affiliated Hospital of Qingdao University, Qingdao, China; ^2^ Department of Endocrinology, Qilu Hospital, Cheeloo College of Medicine, Shandong University, Jinan, China

**Keywords:** low-carbohydrate diet, government expenditure, glycemic control, type 2 diabetes remission, medication withdrawal

## Abstract

**Objective:**

Multiple studies have confirmed that diet restrictions can effectively realize glycemic control and reduce metabolic risks in patients with type 2 diabetes mellitus (T2DM). In 2018, the American Diabetes Association (ADA) and European Association for the Study of Diabetes (EASD) stated that individuals can select a low-carbohydrate diet (LCD) according to their needs and preferences. Owing to the influence of Chinese traditional eating habits, only a small portion of patients in China have achieved their blood glucose goals. As a result, the Chinese government will incur huge expenditures.

**Method:**

This study recruited 134 T2DM participants and randomly assigned them to the LCD group (*n* = 67) or the low-fat diet (LFD) group (*n* = 67). All of the patients had a fixed amount of exercise and were guided by clinicians. After a period of dietary washout, all of the patients received corresponding dietary education according to group. The follow-up time was 6 months. The indicators for anthropometry, glycemic control, and medication application parameters were collected and compared between the two groups.

**Results:**

There were 121 participants who finally entered the study. The proportions of calories from three major nutrients the participants consumed met the requirements of LCD and LFD. Compared with baseline, the pre-postdifferences of body weight, BMI, and several other indicators were significant except for dosages of insulin used in the LCD group and MES in the LFD group. After the intervention, body weight, body weight index (BMI), fasting blood glucose (FBG), postprandial 2-h blood glucose (PPG), and glycosylated hemoglobin (HbA1c) levels in the LCD group decreased significantly (*p* < 0.05) compared with the LFD group. The number of patients using lipid-lowering agents was significant higher in the LCD group and lower in the LFD group. However, there was no significant difference between the two groups for antihypertensive, hormone-replacement, and other agents.

**Conclusions:**

The LCD diet can decrease body weight, glycemic levels, MES, and lipid-lowering agents more than the LFD diet, thus decreasing cost burden in Chinese patients with T2DM. Strict diet control and monitoring are the keys to managing diabetes.

## Introduction

Available evidence confirms that restrictions to the intake of dietary macronutrients can produce considerable improvements in glycemic control and metabolic risk factors in patients with type 2 diabetes mellitus (T2DM) without calorie modification and consequent weight loss (1). However, good compliance is a prerequisite for effective treatment. A joint statement from the American Diabetes Association (ADA) and the European Association for the Study of Diabetes (EASD) in 2018 also noted that low-carbohydrate diets (LCDs) can be applied to manage diabetes and individual selection is advised to adapt the diet preference and metabolic requirements to patients, with the goal of identifying healthy dietary habits that are feasible and sustainable (2). In the late twentieth century, several cross-sectional epidemiological studies and controlled clinical trials proved that decreasing the intake of fat can produce modest spontaneous weight loss (3–6). Therefore, the US government advised the public to increase the intake of carbohydrates and consume all fats sparingly, as exemplified by the Food Guide Pyramid of 1992 (7).

Before the twentieth century, insulin was not available, so scholars advocated the “starvation diet” to treat diabetes mellitus (DM); it was considered the only method to solve hyperglycemia. Subsequently, Dr. Atkins proposed the LCD in 1972 in the book *Dr. Atkins’ Diet Revolution*. At present, there is no unified definition for the LCD/very low-carbohydrate diet (VLCD). Generally speaking, the LCD means that carbohydrates account for 26%–45% of total calories per day, while the VLCD means that carbohydrates account for less than 26% of total calories.

In recent years, increasing evidence suggests that the LCD is effective for weight loss and cardiovascular risk factor reduction (8). ADA and EASD, in 2018, issued the statement that individuals can select the LCD according to needs and preferences (2). A randomized controlled trial in Japan demonstrated that LCD diet for 6 months was more effective and secure than a calorie-restricted diet (CRD) for Japanese patients with T2DM who could not achieve good glycemic control despite repeated CRD adherence (9). A related clinical trial led by China has also shown the dominant position of LCD in glycemic control (10). However, only limited studies in China have paid attention to the drug burden and medication effects score (MES) with the application of the LCD in patients with DM.

Research from China revealed that only 13.3% overweight and 10.1% obese T2DM patients achieved blood glucose, blood pressure, and blood lipid goals (11). The latest Diabetes Atlas published by the International Diabetes Federation (IDF) showed that 10% of the healthcare expenditure of the world was related to diabetes, costing up to US$760 billion. As the country with the largest population of DM patients in the world, China ranked second in the world in healthcare expenditure, at US$109 billion (12). The extremely low eligible rate for the blood glucose standard and the high expenditure in China prompted us to explore a more effective and feasible method to manage DM. Therefore, we explored the efficacy of two DM diets, the LCD and the low-fat diet (LFD), on glycemic control and clinical treatment.

## Materials and Methods

### Subjects

We recruited participants with T2DM in the Affiliated Hospital of Qingdao University. The inclusion criteria were as follows: (1) older than 18 years; had been diagnosed with T2DM (previously diagnosed with typical clinical symptoms; (2) added glycosylated hemoglobin ≥7.0%, added fasting blood glucose ≥7.0 mmol/L, added random blood glucose ≥11.1 mmol/L, or added OGTT 2 h blood glucose ≥11.1 mmol/L); (3) had no change in the hypoglycemic regimen for at least half a month; (4) had volunteered to participate in the study; and (5) were able to communicate, and could give their informed consent. If any of the following conditions were found in the inspection at registration, the selected T2DM patients were excluded from the study: (1) severe complications of DM such as proliferative retinopathy, severe neuropathy, serious kidney disease (serum creatinine level >2.0 mg/dl and/or with microalbuminuria); (2) serious liver disease excluding fatty liver (aspartate aminotransferase and/or alanine aminotransferase levels >100 IU/L) and pancreatic disease; (3) acute heart failure within 3 months or apparent chronic heart failure; (4) active malignancy; (5) pregnancy; (6) serious infectious disease; (7) trauma injury; (8) alcohol dependency; (9) eating nuts regularly (≥4 days/week); (10) food allergy, especially to nuts; (11) having difficulty in chewing nuts (such as individuals with few teeth); (12) not suitable for the study; and (13) having too high a glycemic level and having to increase medications (9, 13).

### Study Design

**Figure 1:**
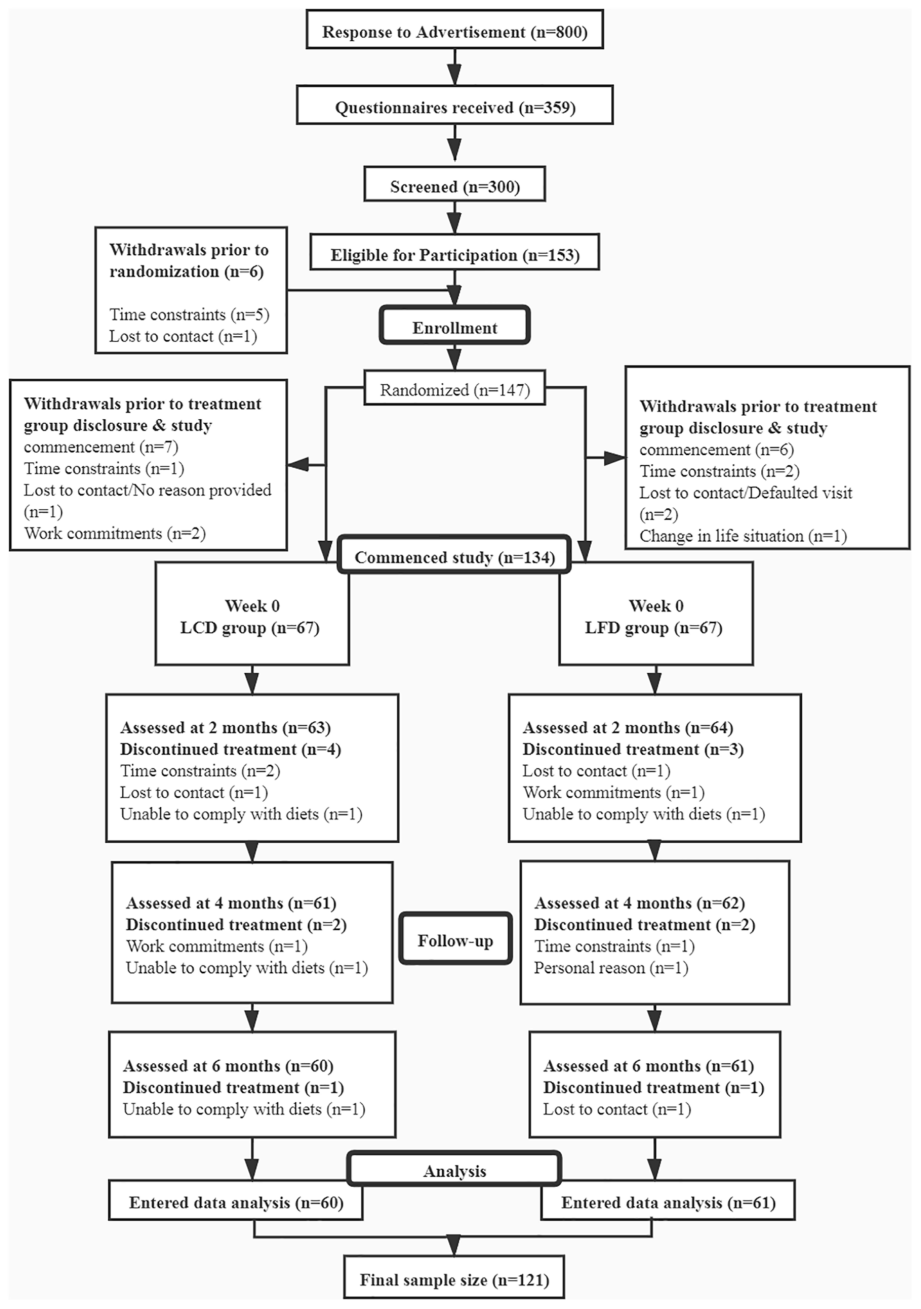
Study flow diagram.

This study was a prospective, open-label, double-arm, randomized controlled trial conducted from March 2019 to December 2020 at the Affiliated Hospital of Qingdao University, China (**Figure 1**). The enlisted participants were randomly assigned to either the LCD group or the LFD group by computer operation. All of the patients went through a period of dietary washout to minimize the influences of background diets before intervention. This study was conducted according to the guidelines of the Declaration of Helsinki and approved by the Ethics Committee of the Affiliated Hospital of Qingdao University (No. QYFYECYJ 2019-007-01).

All of the participants performed moderate intensity aerobic and resistance exercise 3 days a week for 60 min each time under the supervision of professionals. Supervisors had received unified training courses.

### Outcomes

We collected the following at the beginning and end of each intervention: weight, fasting blood glucose (FBG), postprandial 2-h blood glucose (PPG), glycosylated hemoglobin (HbA1c), antiglycemic medications, and medications for other diseases and emerging diseases. The baseline record also included age, gender, height, maximum weight, duration of DM, family history, marital status, and blood pressure. The calculation of exponents, like BMI, will be described later. In addition, adverse events, such as episodes of severe hypoglycemia and ketoacidosis, were also recorded one by one during the intervention periods.

The primary endpoints were weight and variables related to glycemic control. Secondary endpoints included the medication effect score (MES) and other medication changes.

### Sample Size Calculation

Previous research showed that the changes in the MES for 6 months were −0.5 ± 0.5 in the LCD group and −0.2 ± 0.5 in the LFD group (14). Therefore, we calculated 60 patients for each group, with *α* = 0.05 and power = 0.90. Given the sample loss of 10%, the number for each group was 67. Finally, we recruited 67 patients for each group in the study.

### Medication Changes

All of the participants were given medications under the guidance of clinicians who had studied the ADA guidelines (15–17). We tried to avoid making a lot of adjustments to the patients’ drug use within these 6 months. If the glycemic level was stable, the medication could be reduced; if the glycemic level was too high, and the clinician had to increase the medications, the patient would be excluded. MES evaluated the overall usage of various types of antiglycemic agents. Medications were recorded during the whole process. First, the maximum daily dose of each agent was confirmed through retrieval. The maximum daily dose of insulin was defined as 1 unit/kg of the baseline weight, delineating insulin resistance (18). Then, the percentage of the maximum daily dose for each medication was multiplied by an adjustment factor (19, 20), and these outcomes were summed for the final MES (21). Adjustment factors were the reported median absolute decrease in HbA1c for each medication. That is, for metformin and the sulfonylureas, the adjustment factor was 1.5; for insulin, it was 2.5 (**Table 1**). The variety of MES reflected the change of antidiabetic medication dosages that the participants used during the 6 months. Researchers recorded the type and dose of antidiabetic agents at each follow-up.

**Table 1:** Summary of adjustment factor for medications.

Medications	Expected decrease in HA1c with monotherapy (%)	Adjustment factors
Metformin	1.0–2.0	1.50
Sulfonylureas	1.0–2.0	1.50
α-Glucosidase inhibitors	0.5–0.8	0.65
Insulin	1.5–3.5	2.50
TZDs	0.5–1.4	0.95
GLP-1 receptor agonist	0.5–1.0	0.75
Glinide	0.5–1.5	1.00
Pramlintide	0.5–1.0	0.75
DPP-4 inhibitor	0.5–0.8	0.65
SGLT-2 inhibitor	0.5–1.0	0.75

### Biochemical Parameters and Analyses

HbA1c, representing the glucose level for the past 3 months, is the universally accepted standard for the diagnosis and monitoring of diabetes mellitus and the marker for glycemic control and diabetes mellitus outcomes (22). Blood samples were accessed to measure HbA1c at the Affiliated Hospital of Qingdao University and tested by ion-exchange high-performance liquid chromatography using the Tosoh Automated Glycohemoglobin Analyzer (Tosoh Corporation, Yamaguchi, Japan) in the clinical laboratory of the hospital. FBG and PPG were tested by a rapid glucose meter by patients at home or the hospital by collecting fingertip blood.

Adverse events considered in this study included episodes of severe hypoglycemia and ketoacidosis. Hypoglycemic episodes included hypoglycemia with any symptoms accompanied with or without a plasma glucose concentration ≤70 mg/dl (3.9 mmol/L) or only a measured plasma glucose concentration ≤70 mg/dl (3.9 mmol/L). Ketoacidosis was characterized by a plasma ketone body concentration ≥3 mmol/L or positive urine glucose and a ketone body, and a blood-bicarbonate ion <18 mmol/L and/or arterial blood pH <7.3. All of the above factors were considered.

### Anthropometric Measurements

We measured the weight and height of participants by the same measuring device at the Affiliated Hospital of Qingdao University during follow-up. Body mass index (BMI) was calculated as the weight (kg) divided by the height (m^2^).

### Diet Record

Participants kept a detailed diet record, whether on working or rest days, and entered all of the records into the Chinese CDC nutrition calculator V2.63 software, developed by the team of Fei Hua nutrition software, Beijing, China (13). The software can calculate the quantities and distributions of energy from three major nutrients.

### Intervention

The expected major nutrient compositions of the two diets were as follows: LCD group, 14% carbohydrate (the objective was to restrict intake to <50 g/day), 28% protein, and 58% total fat (35% monounsaturated fat and 13% polyunsaturated fat); LFD group, 53% carbohydrate, 17% protein, and 30% total fat (15% monounsaturated fat and 9% polyunsaturated fat). The latter embodied conventional recommendations of current guidelines, and both diets limited saturated fat to <10%. All of the individuals were asked to maintain their basic physical activities during the study. A manual was provided to individuals that contained corresponding recipes, recommendations for food intake at different energy levels, meal plans, and instructions for using and uploading data to the nutrition calculator V2.63 software within the family.

Randomization procedures (sequence generation and allocation concealment) were performed by research associates independent of outcome assessments and intervention delivery. Follow-up was conducted once a month during the intervention. The duration of follow-up and education was around 15 min. The key point was to encourage participants with poor adherence to persevere. Patients who did not meet the diet requirements were excluded. This process was employed throughout the intervention period.

### Statistical Analysis

Data were examined for normality by Shapiro-Wilk test. Statistical analyses were performed by SPSS 26.0 software (SPSS, Inc., Chicago, IL, USA). All graphic productions were processed by Graph-pad Prism 8.0 software (Alliance Development Group, Beijing, China). Sample size calculation was completed by PASS 15.0. For continuous variables, the results were described as the mean ± standard deviation (SD) and compared by Independent-Samples *t*-test. For nonnormally distributed variables, the results were expressed as median (interquartile range) and compared by Mann-Whitney *U* test. For categorical variables, the results were presented as frequency (percentages), compared by Chi-squared test, Fisher’s test, or Fisher’s exact test. The trends and distributions for variables in two groups during the intervention were described by the fold line, scattered point, or front and rear comparison diagram. A *p*-value of <0.05 was considered statistically significant.

## Results

### Study Participant*s*

This study recruited 134 T2DM participants and randomly assigned them to the LCD group (*n* = 67) or the LFD group (*n* = 67). The overall rate of adherence (**Figure 1**) was 90.3% at 6 months; the 6-month adherence rates were 89.6% in the LCD group, and 91.0% in the LFD group (*p* = 0.77). Finally, the data of the 60 in the LCD group and the 61 in the LFD group were analyzed (**Figure 1**). The baseline characteristics of the participants are shown in **Table 2** and did not differ between the two groups. There were no significant differences in each parameter between the two groups. The mean age was 51.45 years. The median height was 170.0 cm, and the median weight was 70.0 kg. Most participants (60.3%) were men. The mean systolic blood pressure (SBP) was 131.49 mmHg, and the mean diastolic blood pressure (DBP) was 79.47 mmHg. Most participants were taking oral anti-glycemic medications; 73.3% in the LCD group and 83.6% in the LFD group. A total of 32.2% were on exogenous insulin and 17.4% were on GLP-1 receptor agonist (GLP-1RA) among all participants. Approximately 40% were on antihypertensives, lipid-lowering agents, hormone-replacement agents, or others (**Table 2**).

**Table 2:** Baseline characteristics of the study population.

	LCD (*n* = 60)	LFD (*n* = 61)	All (*n* = 121)	*p*
**Characteristic**				
Age (years)	49.13 ± 13.06	53.74 ± 13.48	51.45 ± 13.42	0.059^a^
Gender (male)	40 (66.7)	33 (54.1)	73 (60.3)	0.158^b^
Height (cm)	170.0 (162.3–174.3)	170.0 (160.0–174.5)	170.0 (161.5–174.5)	0.514^d^
Weight (kg)	70.0 (65.0–77.8)	71.0 (65.0–80.0)	70.0 (65.0–80.0)	0.441^d^
BMI (kg/m^2^)	24.0 (22.6–27.0)	25.6 (22.7–27.9)	24.5 (22.7–27.3)	0.142^d^
Maximum weight (kg)	78.5 (70.4–90.0)	80.0 (70.00–87.00)	80.0 (70.0–90.0)	0.705^d^
Maximum BMI (kg/m^2^)	27.0 (25.1–30.7)	27.7 (25.7–31.4)	27.3 (25.4–30.9)	0.202^d^
Duration of diabetes (years)	2.0 (0.3–5.0)	4.0 (0.5–9.5)	3.0 (0.3–8.0)	0.133^d^
Family history of diabetes (yes)	40 (66.7)	44 (72.1)	84 (69.4)	0.514^b^
Hypoglycemia (yes)	6 (10.0)	5 (8.2)	11 (9.1)	0.730^b^
Ketoacidosis (yes)	1 (1.7)	0 (0.0)	1 (1.0)	0.496 ^c^
Marital status (married)	59 (98.3)	59 (96.7)	118 (97.5)	1.000^c^
Blood pressure (mmHg)				
Systolic	131.35 ± 12.77	131.62 ± 15.08	131.49 ± 13.92	0.915^a^
Diastolic	80.07 ± 10.01	78.89 ± 12.60	79.47 ± 11.36	0.569^a^
Glycemic control				
Fasting glucose (mmol/L)	8.1 (6.50–12.3)	8.0 (6.3–9.8)	8.0 (6.3–10.4)	0.166^d^
Postprandial 2-h glucose (mmol/L)	11.0 (8.0–14.8)	9.0 (7.6–12.6)	10.0 (7.8–13.5)	0.107^d^
HbA1c (%)	7.7 (7.0–10.1)	7.3 (6.6–8.7)	7.6 (6.8–9.4)	0.099^d^
Medications for diabetes				
Oral antiglycemic medications (yes)	44 (73.3)	51 (83.6)	95 (78.5)	0.169^b^
Intensive insulin therapy (yes)	16 (26.7)	23 (37.7)	39 (32.2)	0.194^b^
GLP-1RA (yes)	14 (23.3)	7 (11.5)	21 (17.4)	0.085^b^
Antiglycemic MES	1.40 (0.9–1.7)	1.60 (1.2–2.1)	1.5 (1.1–2.0)	0.356^d^
Medications for other diseases				
Antihypertensive (yes)	16 (26.7)	22 (36.1)	38 (31.4)	0.265^b^
Lipid lowering (yes)	10 (16.7)	15 (24.6)	25 (20.7)	0.282^b^
Hormone replacement (yes)	3 (5.0)	5 (8.2)	8 (6.6)	0.717^c^
Others (yes)	9 (15.0)	13 (21.3)	22 (18.2)	0.368^b^

Data are available from 121 participants, unless otherwise stated. Data are expressed as mean ± SD or median (interquartile range) or frequency (percentage). All baseline values were not significantly different between diet groups (p > 0.05) by Independent-Samples t-test or Mann-Whitney U test or Chi-square test.

a t-test.

b Chi-square test.

c Fisher’s exact test.

d Mann-Whitney U test.

### Dietary Intake and Compliance

**Figure 2:**
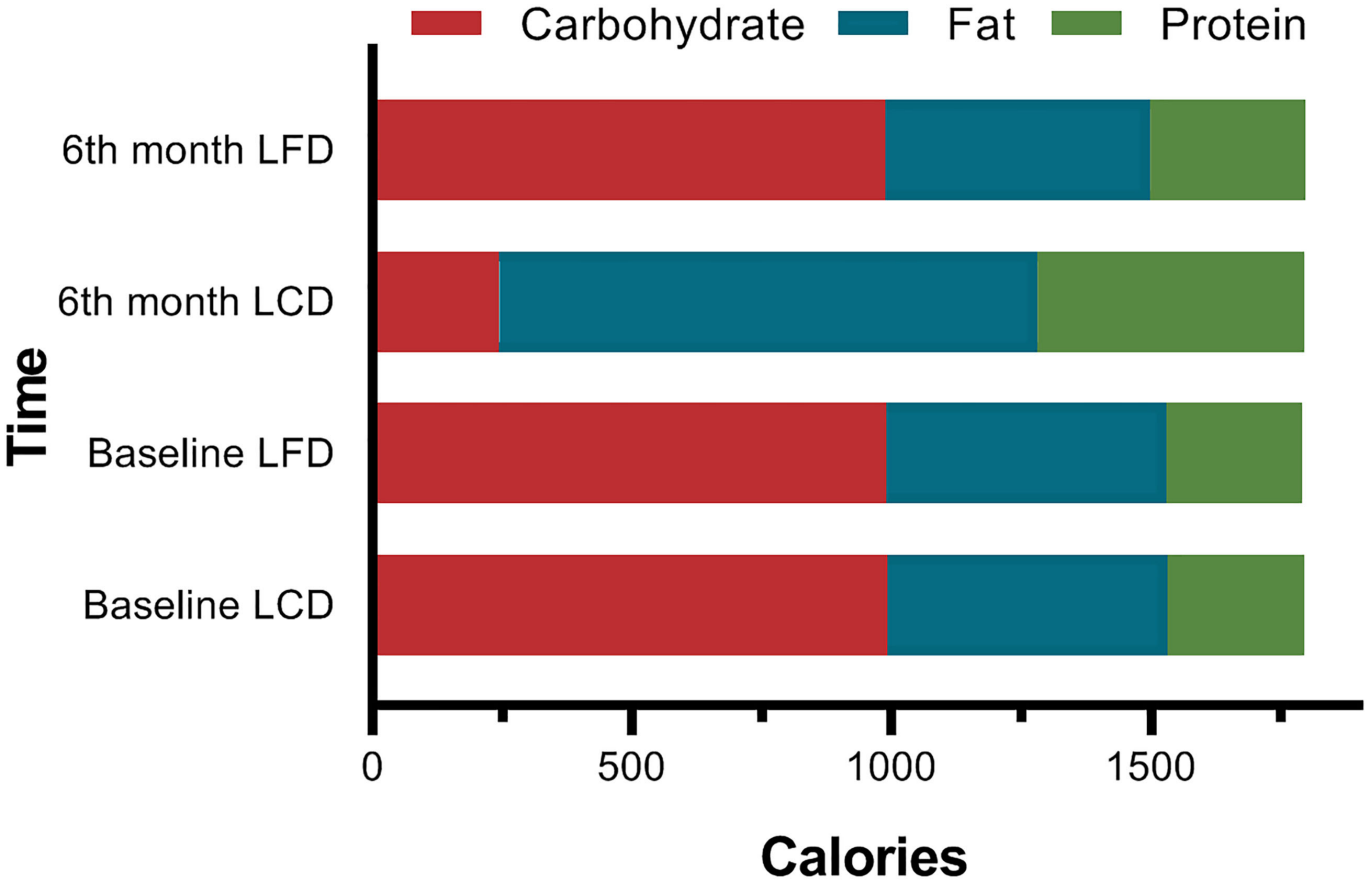
Comparison of the Calories from Three Macronutrients. At baseline, there were no significant differences between the two groups. The percentage of the calories from carbohydrates (13.61%) met the standard of LCD (<14%) in the LCD group, while the 28.38% calories from fat met the standard of LFD.

At the baseline, there were no significant differences in the composition of the diets consumed by participants between the two groups (*p* = 0.927). After 6 months, total calorie intake was similar to the baseline in the LCD and the LFD groups, respectively. In the LCD group, the intake of carbohydrates decreased, and other two nutrients increased after 6 months. In the LFD group, the intake of fat decreased, and that of protein increased, with no change in carbohydrates (**Table 3**). Carbohydrates accounted for 13.61% of energy in the LCD group, and fat accounted for 28.38% in the LFD group, so the abovementioned participants all met the standards of the percentage of calories from major nutrients in different groups (**Figure 2**).

**Table 3:** Comparison of the calories from three macronutrients consumed by the patients.

Variables	LCD (*n* = 60)	LFD (*n* = 61)	*t*	*p*
Baseline				
Total calorie intake/day	1,795.47 ± 195.76	1,792.30 ± 183.50	0.092	NS
Carbohydrate (kcal)	990.70 ± 107.47	988.60 ± 94.57	0.114	NS
Fat (kcal)	539.66 ± 58.75	539.99 ± 58.08	−0.031	NS
Protein (kcal)	265.11 ± 49.99	263.70 ± 44.68	0.163	NS
6th month				
Total calorie intake/day	1,796.20 ± 148.95	1,797.59 ± 153.85	−0.050	NS
Carbohydrate (kcal)	244.45 ± 27.56^*^	987.34 ± 26.55	−151.036	<0.001^**^
Fat (kcal)	1,036.57 ± 36.93^*^	510.12 ± 45.88^*^	69.462	<0.001^**^
Protein (kcal)	515.18 ± 94.56^*^	300.13 ± 92.01^*^	12.679	<0.001^**^

p-value for comparison by Independent-Samples t-test.

^*^There is a significant difference between the baseline and 6th month; ^**^p < 0.001. NS, differences are not significant.

### Anthropometric Indicators

Body weight and BMI also decreased significantly in the LCD group compared with the baseline, but the differences in the LFD group were not significant. Six months later, the data in the LCD group were less than that in the LFD group (*p* < 0.05). Weight loss from baseline values decreased by 4.1 kg [95% CI (−5.5, −2.8) kg; *p* < 0.05] (**Table 4**) in the LCD group. The change in BMI was 1.5 kg/m^2^ [95% CI (−2.0, −1.0) kg/m^2^] in the LCD group.

**Table 4:** Changes in all endpoints after 6 months of intervention.

	LCD (*n* = 60)		LFD (*n* = 61)		*P*_1_	*P*_2_	*P*_3_
	6th month	Change	6th month	Change			
Body weight (kg)	65.0 (60.0, 71.8)	−4.1 (−5.5, −2.8)	70.7 (62.0, 80.0)	−1.0 (−3.7, −0.3)	<0.05^*^	<0.05^*^	0.478
BMI (kg/m^2^)	22.7 (21.5, 24.9)	−1.5 (−2.0, −1.0)	24.7 (22.6, 28.1)	−0.3 (−1.3, −0.1)	<0.001^***^	<0.01^**^	0.438
Glycemic control							
Fasting glucose (mmol/L)	6.2 (5.8, 6.9)	−2.0 (−4.0, −2.1)	6.7 (5.7, 7.6)	−0.7 (−2.2, −0.8)	0.272	<0.001^***^	<0.01^**^
Postprandial 2-h glucose (mmol/L)	7.0 (6.2, 8.0)	−3.7 (−5.6, −3.2)	8.0 (7.0, 9.8)	−1.0 (−2.9, −0.9)	<0.01^**^	<0.001^***^	<0.01^**^
HbA1c (%)	6.0 (5.7, 6.3)	−1.8 (−3.3, −2.0)	6.4 (5.8, 7.2)	−0.6 (−1.6, −0.7)	<0.01^**^	<0.001^***^	<0.001^***^
Medications							
Antiglycemic MES	0.0 (0.0, 0.8)	−1.1 (−1.3, −0.9)	1.6 (1.1, 2.3)	0.0 (−0.1, 0.1)	<0.001^***^	<0.001^***^	0.966
Proportion of cohort that achieved decrease in MES	48 (80.0)	–	21 (34.4)	–	<0.001^***^	–	
≥20% decrease [*n* (%)]	1 (2.1)	–	15 (71.4)	–	–	–	
≥50% decrease [*n* (%)]	47 (97.9)	–	6 (28.6)	–	–	–	
Dosages of insulin used (IU/day)	0.0 (0.0, 0.0)	0.0 (−1.1, 0.1)	0.0 (0.0, 11.0)	0.0 (−3.8, −0.1)	<0.001^***^	<0.05^*^	0.394
Hypoglycemia [*n* (%)]	5 (8.3)		6 (9.8)		0.774		
Ketoacidosis [*n* (%)]	1 (1.7)		0 (0.0)		0.496		
	**Increase**	**decrease**	**increase**	**decrease**			
Other medications change							
Antihypertensive agents	0 (0.0)	6 (10.9)	0 (0.0)	2 (3.3)	–	0.262	
Lipid-lowering agents	5 (8.3)	0 (0.0)	0 (0.0)	7 (11.5)	<0.05^*^	<0.05^*^	
Hormone-replacement agents	0 (0.0)	0 (0.0)	1 (1.6)	0 (0.0)	1.000	–	
Others	3 (5.0)	0 (0.0)	0 (0.0)	1 (1.6)	0.119	1.000	
Emerging diseases [*n* (%)]							
Complications of diabetes	1 (1.7)		5 (8.2)		0.217		
Cancer	0 (0.0)		1 (1.6)		1.000		
Cardiovascular events	0 (0.0)		1 (1.6)		1.000		
Cerebrovascular events	0 (0.0)		0 (0.0)		–		
Other metabolic events	5 (8.3)		1 (1.6)		0.202		
Others^a^	4 (6.7)		1 (1.6)		0.207		

Data are expressed as median (interquartile range) or frequency (percentage), unless otherwise specified. The change data represent the value measured at the end of the 6-month diet therapy minus baseline value, expressed as delta change.

P_1_, differences between groups at 6 months or differences of increased person-times after 6 months; P_2_, differences of change between groups or differences of decreased person-times after 6 months; P_2_, differences between baseline and the 6th month in the LCD group or differences of decreased person-times after 6 months; P_3_, differences between baseline and the 6th month in the LFD group.

^*^p < 0.05; ^**^p < 0.01; ^***^p < 0.001.

a Gastrointestinal disorders, constipation and diverticulitis, esophageal ulcers with Helicobacter pylori infection, nonstudy-related workplace injuries, etc.

### Glycemic Control

#### Comparison and Changing Trends of HbA1c

**Figure 3:**
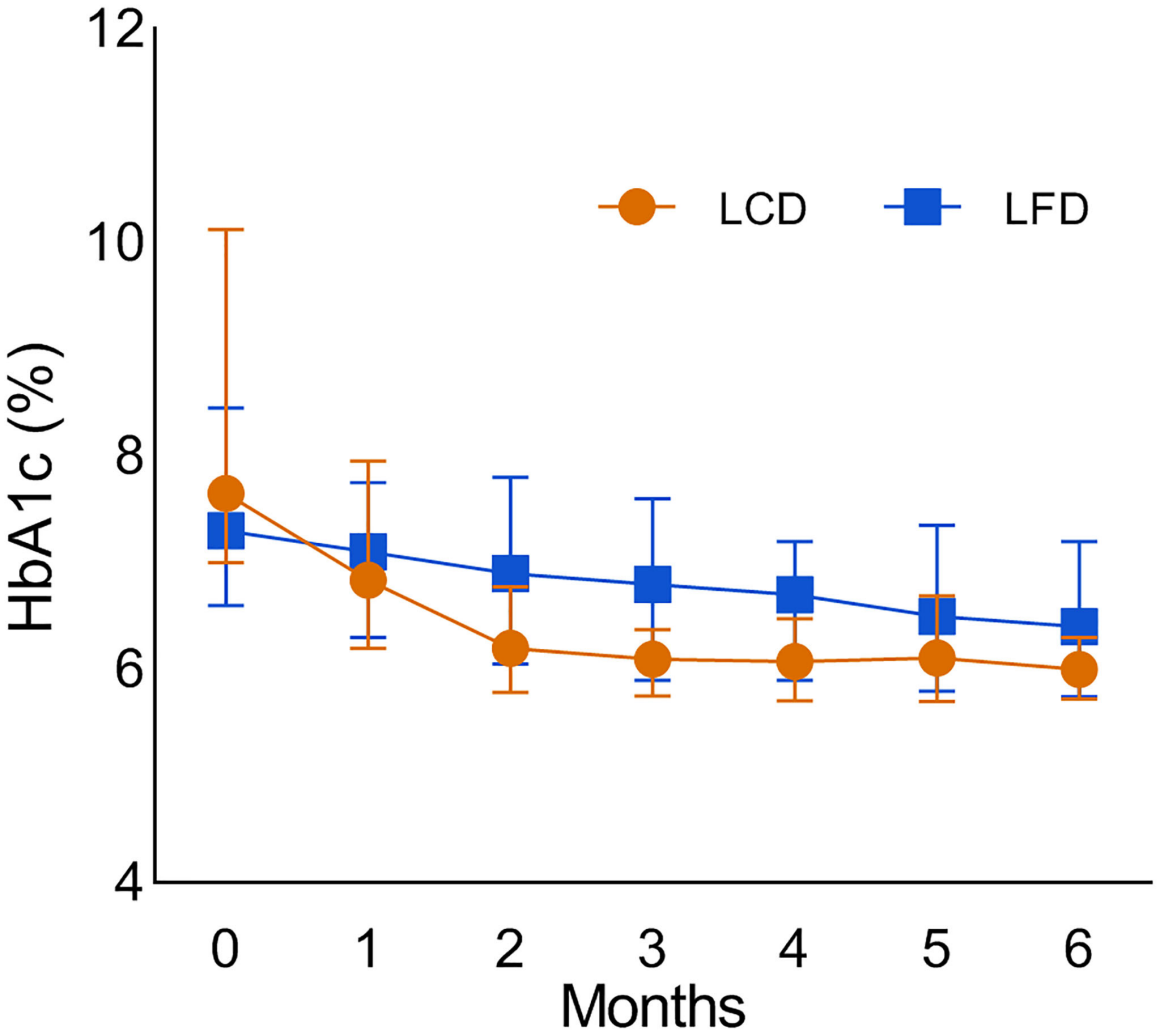
The fold line diagram which describes the changing trends of the HbA1c in the two groups during the intervention. LCD, Low-carbohydrate diet; LFD, Low-fat diet; HbA1c, glycosylated hemoglobin.

Compared with the baseline, HbA1c levels of the two groups significantly decreased [−7.7% (7.0%, 10.1%) versus 6.0% (5.7%, 6.3%) in the LCD group, 7.3% (6.6%, 8.7%) versus 6.4% (5.8%, 7.2%) in the LFD group, *p* < 0.05] (**Tables 3**, **4**), respectively. After the intervention, HbA1c levels in the LCD group decreased significantly [−1.8% (−3.3%, −2.0%) versus −0.6% (−1.6%, −0.7%), *p* < 0.05] (**Table 4**), compared with the LFD group. The fold line diagram describing the changing trends of HbA1c in the two groups during the intervention is shown in **Figure 3**. It can be seen from the diagram that the change of HbA1c in the LCD group decreased significantly for the first 3 months, and then small-amplitude fluctuation occurred after the third month. In contrast, the HbA1c in the LFD group decreased steadily during the intervention, although it also experienced a decline.

#### Comparison and Changing Trends of FBG and PPG

**Figure 4:**
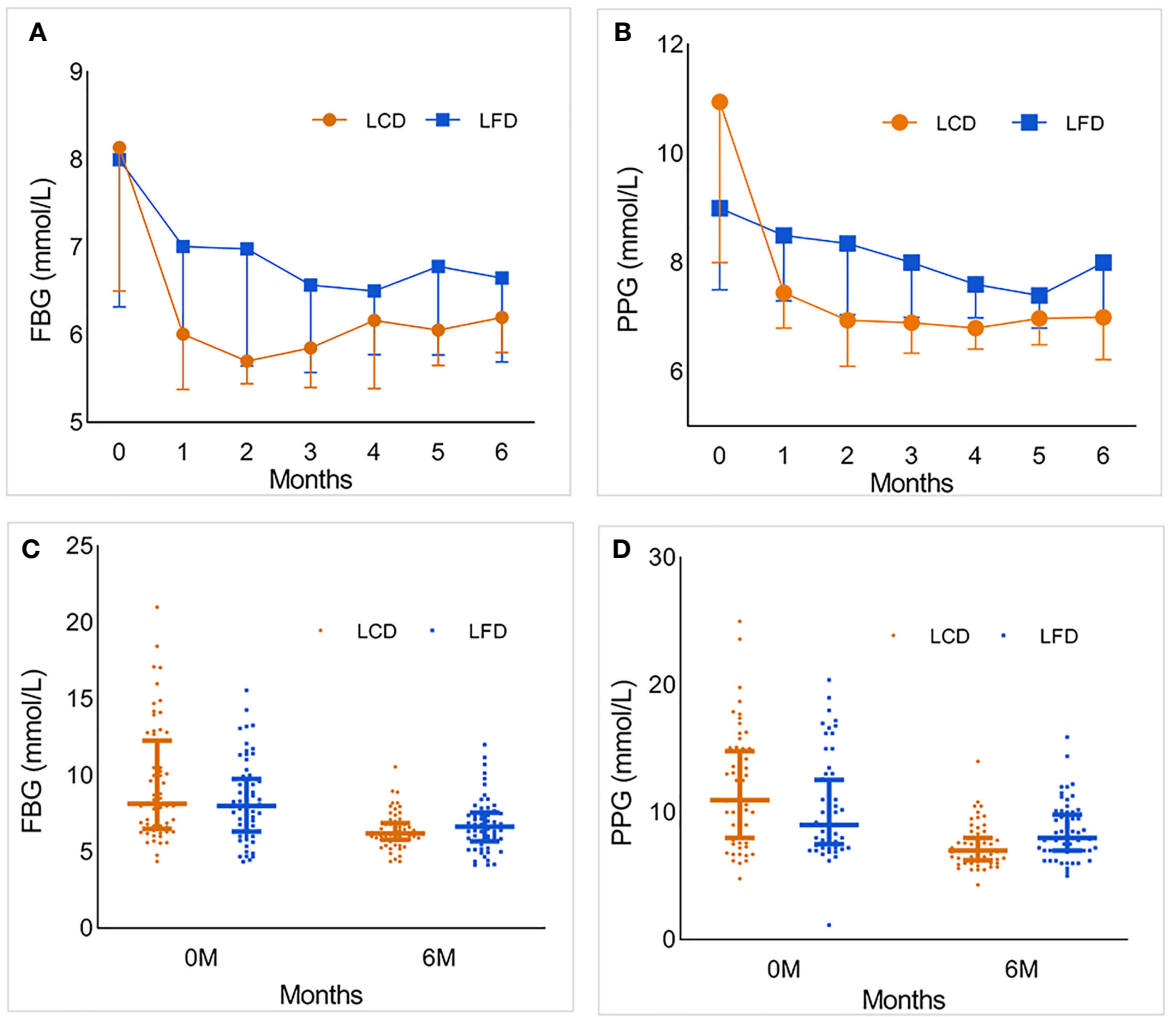
The foldline and scatter diagram which describe the changing trends and distribution of the FBG and PPG of the two groups during the intervention. **(A)** The foldline diagram of FBG in the LCD and LFD group. FBG in both groups decreased significantly in the first month, then gradually become stabilized. And the range in LCD group was more obvious. In the second half of follow-up, FBG fluctuated slightly. **(B)** The foldline diagram of PPG in the LCD and LFD group. The changing trend was similar with FBG. **(C, D)** The scatter diagram of FBG and PPG in the two groups. There was no significant difference in the distribution of FBG and PPG between the two groups at baseline. After 6 months, the level of FBG and PPG in LCD group were lower than LFD group. LCD, Low-carbohydrate diet; LFD, Low-fat diet; FBG, fasting blood glucose; PPG, postprandial 2-h blood glucose.

After 6 months, FBG and PPG in both groups had a significant decline compared with the baseline. During the intervention, FBG changed by −0.7 (−2.2, −0.8) mmol/L in the LFD group, and significantly more in the LCD group (*p* < 0.05) [−2.0 (−4.0, −2.1) mmol/L]. PPG changed by −1.0 (−2.9, −0.9) mmol/L in the LFD group, and significantly more [−3.7 (−5.6, −3.2) mmol/L] in the LCD group (*p* < 0.05). After 6 months, there was a significant difference between the two groups in PPG, but there was no divergence in the FBG. The changing trends and distribution of the FBG and PPG of the two groups during the intervention are shown in the fold line and scatter diagram (**Figure 4**). Both groups showed fluctuation in the FBG. The most notable decline appeared for the first month in the LCD group.

### Medication Changes and Adverse Events

**Figure 5:**
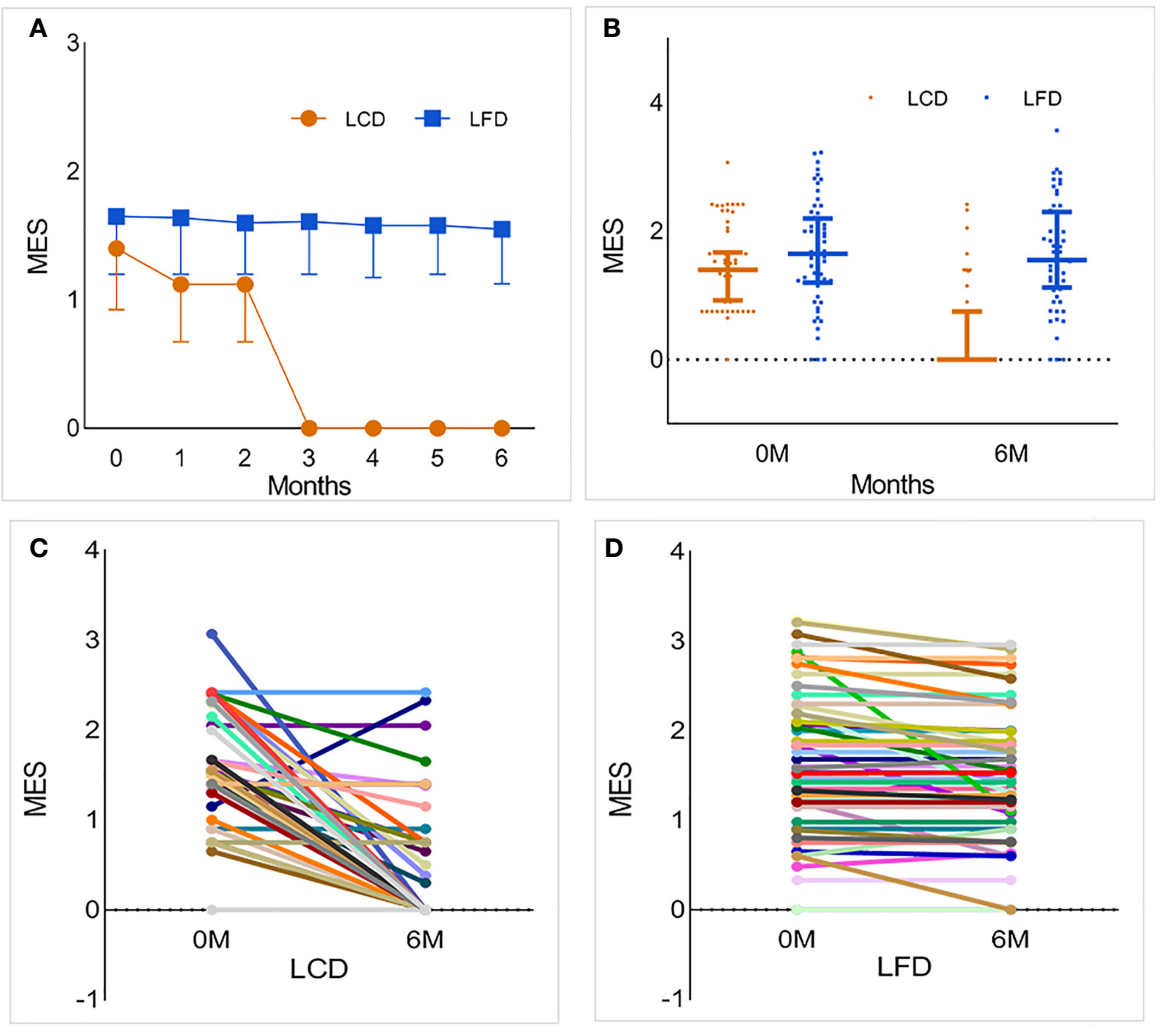
The change and distribution of MES in each group. Most participants in LCD group experienced a period of antiglycemic medications withdrawal. **(A, B)** The foldline and scatter diagram of MES in the two groups. While the blood glucose was stable, the MES of a part of participants in LCD group was zero, which means that they realized medications withdrawal only under the condition of diet restriction. This change was particularly evident after 3 months. However, there was no significant change in LFD group. **(C)** Changes in MES for each participant in the LCD group. Most participants experienced a period of antiglycemic medication withdrawal. **(D)** Changes in MES for each participant in the LFD group. Compared with baseline, most participants had no change or slight decrease in MES. LCD, Low-carbohydrate diet; LFD, Low-fat diet; MES, medication effects score.

At the baseline, medication usage and the antiglycemic MES showed no marked difference in the two groups (*p* = 0.356, **Table 2**). After 6 months, the LCD group experienced significant reductions in the antidiabetic MES [−1.1; 95% CI (−1.3, −0.9)], and the LFD group had no change. Among participants who achieved a decline, more fell by more than 50% compared with the LFD group (*p* < 0.05) (**Table 4**). The change and distribution of MES in each group is shown in **Figure 5A, B**. Moreover, most participants in the LCD group experienced a period of antiglycemic medication withdrawal when all of the behaviors were managed by clinicians (**Figure 5C, D**). There was no case where the patient stopped the medications on their own without stable glycemic levels. The pre-postdifferences of insulin dosages for both groups were not statistically significant. There was no significant difference in the incidence of hypoglycemia or ketoacidosis between the two groups within 6 months before and after intervention (*p* > 0.05).

### The Changes of Other Medications

Five participants in the LCD group began to use lipid-lowering agents, but no one stopped taking it. On the contrary, the number of participants who started and stopped were 0 and 7, respectively, in the LFD group. The difference between the two groups was statistically significant. However, there was no significant difference between the two groups in the 6th month for antihypertensive, hormone-replacement, and other agents (*p* > 0.05, **Table 4**).

### Emerging Diseases

The number of patients in the LCD group who experienced emerging diseases including complications of diabetes, cancer, cardiovascular events, cerebrovascular events, other metabolic events, and other diseases, was 1, 0, 0, 0, 5, and 4, respectively. In the LFD group, the number was 5, 1, 1, 0, 1, and 1, respectively. No significant difference between the two groups was observed after the intervention (*p* > 0.05, **Table 4**).

## Discussion

Measured by the proportion of total daily energy (TDE) from carbohydrates, a carbohydrate-restricted diet was defined as an intake below the lower limit of the acceptable nutrient distribution range for healthy adults (45%–65% TDE). A moderate-carbohydrate diet was defined as 26%–44% TDE from carbohydrates, and a low-carbohydrate diet was defined as 10%–25%, and a very-low-carbohydrate diet was defined as <10% (23). Moreover, 13.61% of TDE in this study was within the restriction of LCD. The nutrition calculator software we used is also the most authoritative nutrition calculation tool.

The Chinese Nutrition Society recommends 250–400 g/day carbohydrates for healthy residents and 45%–60% of TDE (225–300 g/day carbohydrates for a reference 2,000 kcal diet) from carbohydrates for diabetics. The latest dietary guideline of Chinese residents, issued in 2021, pointed out that the dietary structure focuses mainly on cereals, but cereals are mainly refined rice noodles, and the intake of whole grains and miscellaneous grains is insufficient (24). The 13.61% carbohydrate intake set in this study seems to be much lower than the recommended percentage. Because we did not have enough investigations of the LCD in this amount in China, we retained this design in our study. Given the present dietary situation of Chinese residents whose carbohydrate intake is basically higher than in the Western diet, it seemed to be appropriate to perform the study in China.

Our study superiority was the energy matching in advance according to requirements, which removed the potential confounder and enabled metabolic differences between groups to come completely from the differences in the major nutrient ratio. Under the guidance of these diet principles, both groups achieved significant weight loss and BMI decline, but the changes were more obvious in the LCD group. This result is consistent with the results of Wang et al. (13) and Hussian et al. (25). After 3 months, the weight in the two groups gradually tended to become stable, and there was no more obvious change. Thus, we believe that effective control of carbohydrate intake can achieve more significant weight change in the short term.

The famous United Kingdom Prospective Diabetes Study demonstrated that the incidence of clinical complications was significantly associated with glycemia. Each 1% reduction in mean HbA1c was associated with a 21% reduction in risk for any end point related to diabetes, 21% for deaths related to diabetes, 14% for myocardial infarction, and 37% for microvascular complications (26). Effective control of HbA1c can prolong life and improve the quality of life of T2DM patients. In our study, both groups showed an HbA1c decline, but a greater reduction of 1.8% (absolute) occurred in the LCD group. This result keeps pace with that of previous studies, and the decline ratios are equal (9, 13, 14). The possible reason is that T2DM patients with impaired insulin metabolism may experience higher insulin secretion or higher insulin resistance. When in a low-carbohydrate environment, organism will maintain a lower demand or burden on insulin-mediated glucose disposal while remaining a lower carbohydrate and higher fat diet (27). In addition, the extreme restriction of carbohydrates reduces the intestinal absorption of monosaccharide, which leads to a lower blood glucose level and reduces the fluctuation of blood glucose (28).

FBG and PPG are used to diagnose diabetes and monitor blood glucose dynamically in normal times. Their fluctuations more fully reflect the impact of the patients’ diets on blood glucose. Owing to the possible mechanisms mentioned above, FBG and PBG in the LCD group had a sharp decline in the first 2 months, then fluctuated at a lower level. Although the changes of these values do not represent the control of blood glucose, continuous dynamic monitoring can remind patients to adhere to more strict dieting and drug control, which is why the carbohydrate ratio of the LCD group met the standard in our study. The combination of monitoring and electronic technology like nutrition calculator software may be the key to controlling blood glucose effectively for DM patients.

At present, diabetes expenditure ranks first among the personal health care expenditures in the USA; more resources were estimated to be spent on diabetes than any other condition in 2013, and the costs for diagnosis and treatment of DM increased 36 times faster than heart disease (29). The huge economic burden has become a “life-depriving killer” for DM patients. All countries face the problem of medical treatment insurance expenditures rising. Our results show that LCD could control blood glucose effectively and did not increase antiglycemic MES at the same time. We found that MES was decreased in the LCD group but had no change in the LFD group after 6 months. After intervention, the MES between the two groups also had a significant difference. At the same time, the weight and the glycemic level decreased in the LCD group. The same change in the weight and glycemic level was also found in the LFD group, but with no change in the MES. In brief, a lower MES, lower glycemic level, and lower weight coexisted in the LCD group. However, the reduction in medications may have masked the extent of the decline in glycemic levels and weight. This may be related to the release of glucose toxicity, the reduction of insulin resistance, and the reduction of carbohydrate intake. The current results are insufficient to explain the causal relationship between the abovementioned variables, and further study is required to explore the complex relationships between them. Less medication under stable glycemic levels would represent obvious cost savings and ease government expenditure greatly. However, long-term drug withdrawal needs to be combined with strict diet, exercise, and glycemic monitoring. Once patients relax their requirements for diet and exercise, blood glucose will rise again, and islet failure will speed up, which will harm patients.

Compared with the LFD group, participants in the LCD group were neither less nor more likely to experience hypoglycemia or ketoacidosis, suggesting no change in glycemic regulation. This is inconsistent with other study findings demonstrating that lower glucose variability is associated with reduced hypoglycemic risk (30). However, the follow-up of adverse events in this study was not complete, and the sample size was small. Hence, the data related to adverse events should be handled more cautiously and larger studies should be conducted to confirm these results.

Moreover, decreased usage of lipid-lowering agents in the LCD group was observed in our study. However, this does not explain the relationship between the LCD diet and lipid metabolism. The related role is noted as controversial in other literature reviews (23), and we need a larger and longer follow-up for further verification and more relevant indicators, such as triglycerides and cholesterol.

With the further popularization of LCD education and the joint promotion of the Central Food Bank of Pennsylvania and the Geisinger Medical Center, the Fresh Food Farmacy (FFF) project emerged. It embodies the ancient Chinese concept of homology between medication and food and is expected to reduce 80% costs for diabetes treatment (31).

## Strength and Limitations

This study performed energy matching at the beginning of the design to eliminate confounding factors. We observed the effect of LCD on the promotion of the condition of T2DM patients with a larger sample size and different diet habits and traditions in China. This study also had some limitations. First, it lacked a control group of participants who did not receive therapy. Second, we followed up for only 6 months, so the prolonged impact of the LCD was uncertain. A longer observation and a larger sample size are needed to obtain more powerful evidence. In addition, we need to increase the monitoring of blood lipids, uric acid, and other observation indicators related to cardiovascular and cerebrovascular events.

## Conclusion

In brief, the effect of decreasing blood glucose control with the LCD is superior to that of the LFD for Chinese patients with T2DM, yielding a lower MES at the same time. It can reduce body weight, BMI, and lipid-lowering agents. A lower demand or burden on insulin-mediated glucose disposal may play an important role in this process. Strict diet control and monitoring are the keys to managing diabetes.

## Data Availability Statement

The original contributions presented in the study are included in the article/supplementary material. Further inquiries can be directed to the corresponding authors.

## Ethics Statement

The studies involving human participants were reviewed and approved by the Ethics Committee of the Affiliated Hospital of Qingdao University (No. QYFYECYJ 2019-007-01). The patients/participants provided their written informed consent to participate in this study.

## Author Contributions

NY and PL conceived and supervised the overall study. BC, YG, and QW collected the epidemiological and clinical data and summarized all data. YH drafted the manuscript. YH, BC, YG, QW, and PL contributed to the discussion. NY and PL revised the final manuscript. All authors have read and agreed to the published version of the manuscript.

## Funding

This research was funded by the National Natural Science Foundation of China (82070799).

## Conflict of Interest

The authors declare that the research was conducted in the absence of any commercial or financial relationships that could be construed as a potential conflict of interest.

## Publisher’s Note

All claims expressed in this article are solely those of the authors and do not necessarily represent those of their affiliated organizations, or those of the publisher, the editors and the reviewers. Any product that may be evaluated in this article, or claim that may be made by its manufacturer, is not guaranteed or endorsed by the publisher.
